# Translation and cross-cultural adaptation into Brazilian Portuguese of the 35 navigation metrics developed by the Academy of Oncology Nurse & Patient Navigators (AONN+)

**DOI:** 10.31744/einstein_journal/2026AO2089

**Published:** 2026-04-22

**Authors:** Thamyris Pontes Cunha Maia, Mariana Lucas da Rocha Cunha

**Affiliations:** 1 Faculdade Israelita de Ciências da Saúde Albert Einstein Hospital Israelita Albert Einstein São Paulo SP Brazil Faculdade Israelita de Ciências da Saúde Albert Einstein, Hospital Israelita Albert Einstein, São Paulo, SP, Brazil.

**Keywords:** Patient navigation, Oncology nursing, Quality indicators, health care, Benchmarking, Continuity of patient care

## Abstract

**Objective:**

To perform the translation and cross-cultural adaptation into Brazilian Portuguese of the 35 navigation metrics developed by the Academy of Oncology Nurse & Patient Navigators (AONN+).

**Methods:**

This study employed a methodological approach for cross-cultural adaptation. Following authorization from the authors of the original version of the 35 metrics for evaluating navigation programs, the instrument was translated in accordance with established guidelines. A committee of ten experts was formed to review and reach consensus on the translation. The snowball sampling technique was used to recruit 35 judges, including nurse navigator and oncology nurses, with the aim of assessing the applicability of the metrics to the Brazilian context. The data were collected in the first semester of 2025.

**Results:**

The expert committee evaluated whether the metrics achieved semantic, idiomatic, experiential, and conceptual equivalence. Three rounds of evaluation were required to reach a 90% agreement rate. In the subsequent pretest, Brazilian oncology nurses and nurse navigators individually assessed each metric for cultural comprehensibility and applicability. The instrument’s mean content validity index was 0.97.

**Conclusion:**

The 35 metrics were adapted and refined in accordance with the proportions of agreement and content validity index, which reached values consistent with those recommended for adequacy. Therefore**,** the translation enables the use of standardized navigation metrics to measure program outcomes in Brazil.

## HIGHLIGHTS

■ The 35 navigation metrics by AONN+ were cross-culturally adapted.

■ Navigation metrics support the standardization of evaluating navigation results for programs in Brazil.

## In Brief

Maia et al. adapted the metrics developed by the Academy of Oncology Nurse & Patient Navigators (AONN+) into Brazilian Portuguese through a validated cross-cultural process. After expert review and three evaluation rounds with navigators and oncology nurses, the 35 navigation metrics were deemed suitable, enabling the use of standardized navigation metrics to measure program outcomes in Brazil

## Graphical abstract

**Figure:**
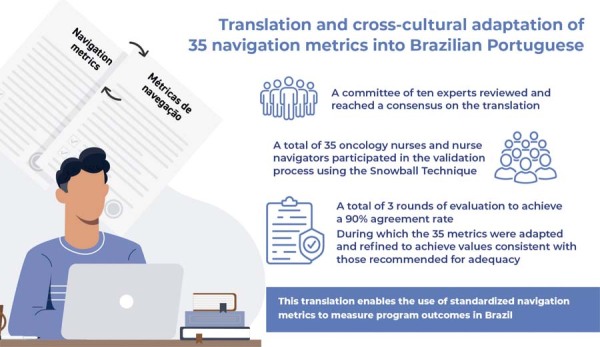

## INTRODUCTION

Currently, according to the Brazilian National Cancer Institute (INCA - *Instituto Nacional de Câncer*), cancer is a public health issue worldwide and one of the leading causes of death in the population. In Brazil, estimates for the 2023-2025 triennium indicate the occurrence of 704,000 new cancer cases, or 483,000 if non-melanoma skin cancers are excluded.^(1)^

Alongside technological advancements, new diagnostic methods and oncology treatment modalities have significantly increased. However, these advances are not equitably accessible to the entire population, leading to disparities in healthcare services and increased mortality and morbidity in oncology patients.^(2)^In this context, physician Harold Freeman played a pivotal role. He was a pioneer in studies and initiatives aimed at understanding health disparities related to the diagnosis, treatment, and follow-up of Black women with cancer.^(3)^

In 1990, at Harlem Hospital in New York, Freeman created the first patient navigation (PN) program aimed at patients with breast cancer. The goal of this program was to eliminate barriers to cancer screening and diagnosis. Following its implementation, the five-year survival rate for patients with breast cancer increased from 39% to 70%. Freeman attributed this improvement to the support provided by PN, as well as access to free and low-cost mammography. These results have prompted significant changes worldwide regarding how oncology services should coordinate and support patient care.^(2,3)^

Today, PN encompasses the entire oncology care continuum rather than only fragmented stages, with the primary aim of eliminating barriers to care. In 2011, new PN concepts were introduced, such as patient-centered care, care coordination, and timely movement of the patients through the healthcare system.^(2)^

The oncology nurse navigator is a nurse specialized in oncology who provides individualized patient care, with primary responsibility for coordinating care and removing barriers so that the patient receives integrated care and moves smoothly and continuously through the healthcare system. Their work must be grounded on evidence-based practice and the nursing process, in addition to knowledge of public policies, social rights, and how the health system functions.^(4,5)^

Nurse navigators are also responsible for patient education and empowerment, enabling active participation in care and promoting shared decision-making. This professional serves as the link between the patient and the healthcare service and as the point of contact for all support areas and the multidisciplinary team.^(4,5)^

In Brazil, navigation is still recent, but some of its principles have already been implemented through previous initiatives in the country. In 2012, Law 12,732 was enacted, establishing a 60-day limit for initiating cancer treatment from the date of pathological confirmation.^(6)^In 2019, this law was amended by law 13,896, which also set a 30-day limit for performing diagnostic tests related to cancer.^(7)^

The first national programs were established in 2014, being concentrated mainly in the Southeast region of Brazil. In 2016, the National Supplementary Health Agency (ANS - *Agência Nacional de Saúde Suplementar*) published the OncoRede project, a manual proposing a set of actions to reorganize and integrate oncology care in the private health sector. It includes a chapter dedicated to navigation, describing its benefits and providing guidelines for implementing a program.^(8)^

In 2022, the federal government established guidelines for a National Navigation Program within the Brazilian Unified Health System (SUS - *Sistema Único de Saúde*) for individuals with breast cancer.^(9)^ In the following year, the SUS National Policy for Cancer Prevention and Control and the National Navigation Program for Individuals Diagnosed with Cancer were established (Law 14,758/2023). This policy calls for navigation programs to eliminate access barriers throughout the oncology care continuum, from primary healthcare to specialized services.^(10)^

It is important to emphasize that neither policy distinguishes between clinical navigators or non-professional navigators.^(9,10)^ A major milestone for PN in Brazil was the regulation of the role of nurse navigators by the Federal Nursing Council (COFEN - *Conselho Federal de Enfermagem*) through Resolution 735/2024.^(11)^

Currently, the literature provides robust evidence demonstrating the benefits for oncology patients followed by a navigator. Studies show that navigated patients experience more effective care coordination and achieve better outcomes in quality of life, psychosocial screening, and symptom management. PN significantly affect clinical outcomes and organizational performance, with higher screening rates, reduced time to diagnosis and treatment initiation, and lower rates of hospital admissions and emergency visits.^(12-25)^

Over recent decades, navigation programs have grown exponentially. PN influences key components recommended by quality certification programs, such as excellence in care, resource management, and financial return. Therefore, to enable a critical assessment of overall outcomes and improve processes throughout the oncology care continuum, it is essential to use evaluation parameters and stratify results through quality metrics.^(26-28)^

The Academy of Oncology Nurse & Patient Navigators (AONN+), founded in 2009, is the largest global organization focused on PN. Its mission is to advance the role of navigation in supporting oncology patients throughout the care continuum and to develop evidence-based practices that enhance care coordination and access for these patients.^(29)^

In 2015, the AONN+ initiated discussions on the development of navigation metrics, emphasizing the need to reflect the quality criteria required for certification, as well as the principles of value-based healthcare.^(30)^In 2016, researchers categorized the effects of navigation outcomes into three main areas: patient experience (PE), return on investment (ROI), and clinical outcomes (CO).^(31)^

In the same year, the AONN+ funded a project to develop metrics to evaluate outcomes and ensure the sustainability of navigation programs. Initially, a task force of ten experts conducted a literature review of approximately 300 documents. The final product was the 2017 publication of 35 metrics validated using a Likert scale to assess their strength of evidence.^(32)^

The 35 metrics were classified into eight domains considered essential by the AONN+: 1) Community Outreach and Prevention, 2) Coordination of Care/Care Transition, 3) Patient Advocacy/Patient Empowerment, 4) Psychosocial Support Services/Assessment, 5) Survivorship/End of Life, 6) Professional Roles and Responsibilities, 7) Operations Management/Organizational Development/ Healthcare Economics, and 8) Research/Quality/Performance Improvement. These domains were subsequently grouped into the three areas of impact: PE, ROI, and CO.^(32)^

In Brazil, given the relatively recent implementation of navigation programs, standardizing metrics for evaluating outcomes still needs further development and consolidation. Translating and adapting into Brazilian Portuguese the 35 navigation metrics developed by the AONN+ will enable oncology navigation programs in the country to use these tools to measure outcomes, improve quality and comprehensiveness throughout the oncology care continuum, and facilitate benchmarking comparisons across services.

## OBJECTIVE

To perform the translation and cross-cultural adaptation into Brazilian Portuguese of the 35 navigation metrics developed by the Academy of Oncology Nurse & Patient Navigators.

## METHODS

This is a methodological study involving the translation and cross-cultural adaptation into Brazilian Portuguese of the 35 navigation metrics published by the AONN+ in 2017. We followed the methodology established by Beaton et al.,^(33)^ which comprises six stages: 1) Translation, 2) Synthesis of translations, 3) Back-translation, 4) Expert committee review, 5) Pretesting, and 6) Submission of the final translated and adapted version to the original instrument authors.^(33)^

Authorization for the translation and cross-cultural adaptation into Brazilian Portuguese was obtained from Strusowski, et al,^(32)^ the lead author of the document and head of the AONN+ metrics project. The current recommendation for the use of the AONN+ metrics was also discussed with the author. The organization maintains its recommendation of the original 35 evidence-based metrics published in 2017, allowing each program to independently analyze its own needs and service characteristics and to select the metrics applicable to its context.

For this study, the Beaton et al.^(33)^ methodology was adapted in some steps. In stage 2 (synthesis), the process was conducted by two observers (the researcher and the adviser). In stage 4 (expert committee review), the translators did not take part in the analysis.

For stage 1 (translation), two professional Brazilian translators fluent in English were selected. The first translator holds a degree in Languages (Portuguese/English) and has experience in translation and text revision in the editorial field. The second translator holds a degree in Veterinary Medicine and specializes in medical translation. Two independent reports were generated: Translation 1 (T1) and Translation 2 (T2). Stage 2 (synthesis of translations) was performed by the two observers, based on T1 and T2, to identify discrepancies and make necessary adjustments. This resulted in the T12 report.

Stage 3 (back-translation) involved translating the T12 version back into the original language to check the similarity with the original document. Two independent back-translations were produced: Back-translation 1 (B1) and Back-translation 2 (B2). The back-translators were professionals with English as their native language, fluent in Brazilian Portuguese, and with no background in the field of study. Stage 4 involved an expert committee to evaluate all versions (T1, T2, T12, B1, and B2), validate the content of T12, and produce a final report (FR) for pretesting.

The committee was formed according to the Strusowski et al.^(32)^ criteria, except for the inclusion of the translators. Committee members were selected after an extensive review of their curricula vitae on the Lattes Platform of the Brazilian National Council for Scientific and Technological Development (CNPq - *Conselho Nacional de Desenvolvimento Científico e Tecnológico*), considering oncology nurses, professional experience, and methodological expertise.^(34)^ The committee comprised ten judges fluent in the target language (English), categorized into two groups: Group 1 (n=2) included oncology nurses with clinical and scientific expertise in the field of study, both with more than ten years of professional experience; and Group 2 (n=8) included professionals with methodological expertise in cross-cultural adaptation, holding doctoral (n=7) or master’s (n=1) degrees and an initial degree in nursing.

An invitation email explaining the objectives of the study was sent to selected experts. Those who agreed to participate were directed to the Research Electronic Data Capture (REDCap) platform to sign the electronic Informed Consent Form (e-ICF). Only after consent did they gain access to the data collection instrument. The instrument presented the 35 AONN+ metrics in the following versions: original, T1, T2, T12, B1, and B2. Experts analyzed all six versions and, considering T12, indicated “agree” or “disagree,” with an open field for comments and suggestions when “disagree” was selected.

For the pretest stage, 35 nurses were selected to validate the FR content. The sample included: 1. Nurse navigators with at least one year of experience, and 2. Oncology specialist nurses with at least ten years of experience, all currently practicing. A snowball sampling technique was used, starting with one participant who referred to another, and so on. This technique is used to recruit key research elements and sources.^(34)^Participants who agreed to participate signed the e-ICF before accessing the data collection instrument.

The instrument consisted of two parts: Part A. Questionnaire on the participants’ professional profiles, and Part B. FR containing the 35 metrics validated by the expert committee. Each metric was individually evaluated for content and applicability to the Brazilian context using a Likert scale: 4 = strongly agree, 3 = agree, 2 = disagree, and 1 = strongly disagree. Items rated 1 or 2 required comments and suggestions for adjustments. The time to complete the instrument was approximately ten minutes.

For the expert committee, content validity was analyzed using the percentage agreement (PA), with a minimum threshold of 90% agreement per metric. For the pretest, participant profiles were analyzed, and content validity was calculated using the content validity index (CVI), both total and per metric, with a minimum acceptable CVI of 0.80.^(35-45)^

All ethical standards for research involving human participants were rigorously followed in accordance with Resolution 466/2012. The study was approved by the Research Ethics Committee (REC) of the *Hospital Israelita Albert Einstein*, CAAE: 80309924000000071; # 7.124.864. Data collection occurred from January to June 2025.

## RESULTS

The expert committee evaluated all versions produced (T1, T2, T12, B1, and B2), based on the original AONN+ document. After reviewing all versions, the judges individually assessed whether the T12 synthesis met the semantic, idiomatic, experiential, and conceptual equivalences. Judges who disagreed provided comments and suggestions for adjustments, which were analyzed in subsequent rounds. A total of three rounds were necessary to reach the minimum 90% agreement for each metric.

The committee consisted of ten judges fluent in the target language (English): 70% were nurses with doctoral degrees, 10% held master’s degrees – all with methodological expertise in cross-cultural adaptation – and 20% were oncology nurses with PN experience.

In the first round, 19 metrics reached a PA ≥90%, while 16 metrics did not achieve the required level of agreement (Table 1), requiring a second round for re-analysis.

**Table 1:** Metrics not achieving a minimum percentage agreement of 90% in the first round

Metric number	Metric	Number of participants agreeing	% agreement
Domain: Community Outreach and Prevention			
1	Cancer screening - referral for diagnostic investigation	7	70
4	Population disparities in screening programs	4	40
Domain: Coordination of Care/Care Transition			
5	Adherence to treatment	8	80
10	Patient guidance	6	60
12	From diagnosis to initial oncology appointment	8	80
Domain: Patient Advocacy/Patient Empowerment			
13	Patient goals	8	80
15	Learning style selection	7	70
Domain: Psychosocial Support Services/Assessment			
16	Psychosocial distress screening	8	80
Domain: Survivorship/End of Life			
18	Survivorship care plan	7	70
20	Social support referrals in survivorship appointment	8	80
21	Palliative care referrals	8	80
Domain: Professional Roles and Responsibilities			
23	Annual review of the fundamental competencies of oncology navigators	7	77.78
Domain: Operations Management/Organizational Development/ Healthcare Economics			
30	Emergency visits	8	80
Domain: Research/Quality/Performance Improvement			
33	Navigation program validation based on community needs assessment	8	80
34	Patient transitions since arrival	8	80
35	Diagnostic investigation	7	70

The main changes implemented consisted of refinements to terms commonly used in Brazil, thereby enhancing cultural and linguistic comprehension (Table 2), as well as adapting references to United States policies to corresponding Brazilian policies. In summary, the necessary adjustments were subtle, consisting of semantic and grammatical modifications, except for a few culturally significant terms that are crucial in oncology. It was recommended to replace the term “*triagem*” (detection) with “*rastreamento*” (screening), along with an updated definition. In the synthesized version, the description of “screening” was more generic; therefore, the judges proposed a technical explanation. The definition was revised based on a globally adopted manual for early cancer detection.

**Table 2:** Adjustments suggested in the first round

Version T12	Adaptation
Detection (*triagem*)	Screening (*rastreamento*)
Social support referrals	Multidisciplinary service referrals
Emergency department	Emergency service
Abnormal finding	Altered diagnostic examination
Chemotherapy, surgery, radiation, endocrine therapy, and biotherapy	Medication, radiation, and surgery

Metric 4 presented the lowest agreement, with a PA of 40%. Originally, this metric introduced the concept of population disparity as defined by the National Institute on Minority Health and Health Disparities (NIMHD). The judges indicated the need for an appropriate definition for the Brazilian context, and the adjustment was made based on a national cancer prevention and control policy.

The instrument sent for the second round comprised 16 metrics for reevaluation by the judges. Of these 16 metrics, six did not reach the minimum PA of 90%, requiring a third round.

The main point of concern remained metric 4 (Population Disparity). For this metric, the conceptual foundation was expanded beyond the previously cited policy to include Brazil’s Organic Health Law, thereby strengthening both its conceptual robustness and applicability to the Brazilian context. In metric 16, the ideal timing for psychosocial screening was described in detail according to the recommendations of the National Comprehensive Cancer Network (NCCN). The judges suggested that domain 5 (Survivorship and End of Life) be split into two separate domains: one for survivorship and one for end of life. As a result, metrics 18, 19, and 20 were assigned to the survivorship domain, and metric 21 to the end-of-life domain. The judges also recommended replacing the term “survivorship appointment” with “post-oncological treatment”. In the third round, the remaining six metrics reached the minimum PA of 90%. At the conclusion of the committee’s validation process, among the 35 metrics evaluated, 20 achieved a PA of 90% and 15 achieved a PA of 100%.

For pretesting, the instrument containing the FR version with all 35 metrics was sent to oncology nurses and nurse navigators in Brazil. Data collection lasted two weeks, resulting in a final sample of 35 participants: 51.4% nurse navigators and 48.6% oncology nurses, with a mean professional experience of ten years. Among the participants, 71.4% were familiar with the AONN+, but 68.6% were unaware of the 35 metrics standardized by the organization in 2017 (Table 3).

**Table 3:** Profile of pretesting participants and their prior knowledge of the 35 AONN+ metrics

Variable	n=35
Nurse - n (%)	
Navigator	18 (51.4)
Oncologist	17 (48.6)
How long have you been practicing?	
Mean	10.89 (9.67)
Are you familiar with the AONN+? - n (%)	
No	10 (28.6)
Yes	25 (71.4)
Are you familiar with the 35 metrics standardized by the AONN+? - n (%)	
No	24 (68.6)
Yes	11 (31.4)

The participants evaluated each metric in the FR individually in terms of its content and applicability to Brazil, with space for comments. All metrics achieved an individual CVI >0.90 (Table 4), concluding data collection in a single round. To assess the instrument as a whole, the mean CVI was calculated by summing the CVIs of all items and dividing by the total number of items evaluated (n=35). The mean CVI for the instrument was 0.97.

**Table 4:** Individual content validity index for each metric and the total for the instrument

Metric number	Metric	Number of participants agreeing	CVI
Domain: Community Outreach and Prevention			
1	Cancer screening - referral for diagnostic investigation	35	1.00
2	Cancer screening	34	0.97
3	End of diagnostic investigation	35	1.00
4	Population disparities in screening programs	34	0.97
Domain: Coordination of Care/Care Transition			
5	Adherence to treatment	35	1.00
6	Barriers to care	34	1.00
7	Interventions	34	1.00
8	Orientation on clinical trials	34	0.97
9	Clinical trial referrals	34	0.97
10	Patient education	32	0.97
11	From diagnosis to initial treatment	34	0.97
12	From diagnosis to initial oncology appointment	33	0.94
Domain: Patient Advocacy/Patient Empowerment			
13	Patient goals	34	0.97
14	Support for caregivers	34	0.97
15	Identification of learning styles	34	0.97
Domain: Psychosocial Support Services/Assessment			
16	Psychosocial distress screening	35	1.00
17	Social support referrals	33	0.97
Domain: Survivorship/End of Life			
18	Survivorship care plan	33	0.97
19	Transition from treatment to survivorship	34	0.97
20	Multidisciplinary referrals in post-oncological treatment follow-up appointments	34	0.97
21	Palliative care referrals	33	0.94
Domain: Professional Roles and Responsibilities			
22	Navigation knowledge at the time of orientation	34	0.97
23	Annual review of the fundamental competencies of oncology navigators	34	0.97
Domain: Operations Management/Organizational Development/ Healthcare Economics			
24	30-, 60-, and 90-day readmission rates	34	0.97
25	Operational navigation budget	31	0.91
26	Navigation caseload	34	0.97
27	Referrals for revenue-generating services	34	0.97
28	No-show rate	31	0.91
29	Patient retention through navigation	33	0.94
30	Emergency visits	33	0.97
31	Emergency admissions by number of patients undergoing chemotherapy	33	0.94
Domain: Research/Quality/Performance Improvement			
32	Patient experience or patient satisfaction survey results	35	1.00
33	Navigation program validation based on community needs assessment	32	0.91
34	Patient transition since institutional admission	34	0.97
35	Diagnostic investigation	33	0.97
	Overall		0.97

CVI: content validity index.

The final translated and adapted version of the instrument (Table 1S, Supplementary Material) was sent to the original authors, with their approval.

## DISCUSSION

The final instrument methodologically achieved the required CVI of 0.97 in the first round of pretesting. At this stage, however, participants provided qualitative suggestions to improve clarity and language without changing the validated conceptual content. The suggestions and comments were extremely valuable for contextualizing the metrics to the Brazilian healthcare setting.

For metric 4, which defines equity and disparity in the context of the Brazilian SUS, participants suggested expanding the concept to include supplementary health care.^(46,47)^ Metrics 8 and 9 address referrals to clinical trials. Given the lower incidence of clinical trials in Brazil compared with the United States, participants noted that these metrics may have limited applicability in the national context.

For metrics 18 and 19, which address care planning and transition to survivorship, it was recommended that the term “curative intent” be removed. This change aligns with the American Society of Clinical Oncology (ASCO) definition of survivorship care, which applies regardless of curative treatment intent and may also encompass maintenance care.^(48)^For metric 21, participants suggested a clearer definition of palliative care, emphasizing that it includes symptom relief and promotion of quality of life, regardless of disease stage. Palliative care may therefore be relevant at diagnosis or during active treatment.^(49)^

Metrics 22 and 23 address navigator training on admission and periodically throughout service delivery. Judges requested clarification of which type of navigator was being referenced – nurse navigators or non-professional navigators. To address this, the legend was updated to specify that, in nurse PN programs, reference will be made to Resolution 735/2024 of the Federal Nursing Council (COFEN), which regulates nurse navigator practice.^(11)^

Metric 25 traditionally describes the monthly operational budget required to sustain a navigation program but does not consider the outcomes generated by these costs. Participants recommended that this metric explicitly link costs to program outcomes, such as clinical results, patient experience, and organizational performance. For metric 27, which counts referrals to revenue-generating services per professional, participants suggested that referral quantification should be aggregated at the program level rather than attributed to individual navigators. For metric 28, no-show rate, the definition of no-shows was expanded to include missed treatment visits (*e.g.*, infusions and radiation therapy) in addition to missed medical appointments.

Metric 29, patient retention through navigation, prompted discussion on how navigation programs in Brazil might contribute to patient retention within supplementary health care, where financial considerations are currently the main determinant of access.

Several terms and definitions were refined to improve semantic accuracy. Metrics 11 and 12, “from diagnosis to initial treatment” and “from diagnosis to initial oncology appointment” were revised to “time elapsed in days from diagnosis to treatment initiation” and “time elapsed in days from diagnosis to first oncology appointment”. Metric 17, “social support referrals” was changed to “referrals to social support networks”. Metric 31, “chemotherapy” was replaced with “antineoplastic therapy”. Metric 33, “monitor” was revised to “monitoring”. Metric 35, the specification “number of working days between the date of the diagnostic exam and the date of the anatomopathological result” was removed, leaving only “calendar days.”

## CONCLUSION

The 35 metrics proposed by the AONN+ were successfully adapted into Brazilian Portuguese. After three rounds of expert evaluation, all metrics achieved satisfactory agreement levels and content validity index values. The validation of these metrics will enable navigation programs to measure outcomes and compare services, fostering critical analysis of healthcare quality and reflection on patient navigation program results in terms of added value.

Nurse navigators must recognize their role not only in data collection but also in interpreting and applying results to improve patient outcomes. Implementing metrics in navigation services strengthens professional practice, defines value through measurable outcomes, and supports program sustainability. The standardization of navigation metrics also enables the analysis of outcomes and reflects fundamental aspects of cancer care pathways, highlighting gaps and informing necessary adjustments to care coordination. Thus, navigation programs and nurse navigators reaffirm their commitment to promoting more equitable and safe health care.

## SUPPLEMENTARY MATERIAL

SUPPLEMENTARY MATERIAL
