# Supplementary material for: Translation and cross-cultural adaptation into Brazilian Portuguese of the 35 navigation metrics developed by the Academy of Oncology Nurse & Patient Navigators (AONN+)
Source: Einstein (Sao Paulo). 2026 Apr 22;24:eAO2089. doi: 10.31744/einstein_journal/2026AO2089 (PMC13128247; doi:10.31744/einstein_journal/2026AO2089)
Supplement: SUPPLEMENTARY MATERIAL [file 2317-6385-eins-24-eAO2089-suppl01.pdf]

SUPPLEMENTARY MATERIAL

Translation and cross-cultural adaptation into Brazilian Portuguese of the 35 navigation metrics developed by the Academy of Oncology Nurse & Patient Navigators (AONN+)

Thamyris Pontes Cunha Maia, Mariana Lucas da Rocha Cunha

DOI: 10.31744/einstein_journal/2026AO2089

Table 1S. Versão em português das 35 métricas de navegação da Academy of Oncology Nurse & Patient Navigators (AONN+)

**Domínios das métricas da AONN+ adaptados para o português do Brasil e com validade de conteúdo**		
1 - Alcance Comunitário e Prevenção		
2 - Coordenação/Transição de Cuidados		
3 - Empoderamento/Defesa do Paciente		
4 - Avaliação/Apoio Psicossocial		
5 - Sobrevivência		
6 - Cuidados Paliativos/Fim de vida		
7 - Funções e Responsabilidades Profissionais		
8 - Gestão de Operações, Desenvolvimento Organizacional e Economia da Saúde		
9 - Pesquisa, Qualidade e Melhoria de Desempenho		
**Áreas de impacto das métricas da AONN+ adaptados para o português do Brasil e com validade de conteúdo**		
1 - Experiência do Paciente (EP)		
2 - Resultados Clínicos (RC)		
3 - Retorno do Investimento (RDI)		
**Métricas por Domínio**		
**Domínio 1: Alcance Comunitário, Prevenção**		
Número e Métrica	Definição	Área de Impacto:<br>Experiência do Paciente (EP)<br>Resultados Clínicos (RC)<br>Retorno do Investimento (RDI)
1 - Rastreamento do câncer*<br>Encaminhamento para investigação diagnóstica	Número de pacientes navegados por trimestre com exame de rastreamento alterado encaminhados para investigação complementar	EP, RC e RDI
2 - Rastreamento do câncer	Número de participantes em eventos de rastreamento de câncer e/ou aumento percentual do rastreamento de câncer.	EP e RC
3 - Conclusão da investigação diagnóstica	Número de pacientes navegados por mês/trimestre com exames de rastreamento alterados que completaram a investigação diagnóstica.	RC e RDI
4 - Disparidade Populacional† nos programas de rastreamento	Número de indivíduos por trimestre em programas comunitários de rastreamento, analisando a participação de grupos historicamente vulneráveis.	EP e RC

* Definição de rastreamento do câncer: De acordo com a Organização Mundial da Saúde (2020)rastreamento caracteriza-se pela aplicação de testes em pessoas assintomáticas, em uma população-alvo definida, com o objetivo de reduzir a morbimortalidade atribuída a uma doença específica. † Disparidade Populacional: No Brasil são princípios do Sistema único de Saúde(SUS) a universalidade e equidade, onde a saúde é um direito de todas as pessoas, independentemente de sexo, raça, ocupação ou outras características sociais ou pessoais. O princípio da equidade traz a consciência de que as pessoas são diferentes e deve-se garantir respeito às diversidades étnico-raciais, culturais, sociais e religiosas, assim como aos hábitos e culturas locais, considerando as diferentes vulnerabilidades, com a finalidade de reduzir as iniquidades em saúde (Lei orgânica de saúde, nº 8.080, de 19 de setembro de 1990 e a Política Nacional de Prevenção e Controle do Câncer no âmbito do Sistema Único de Saúde- SUS/Programa Nacional de Navegação da Pessoa com Diagnóstico de Câncer, Nº 14.758,de 19 de dezembro de 2023).

continue...

...Continuation

Domínio 2: Coordenação/Transição de Cuidados		
Número e Métrica	Definição	Área de Impacto: Experiência do Paciente (EP) Resultados Clínicos (RC) Retorno do Investimento (RDI)
5 - Adesão ao Tratamento	Porcentagem de pacientes navegados por trimestre que aderem aos tratamentos propostos.	RC e RDI
6 - Barreiras ao cuidado*	Número e lista de barreiras específicas ao cuidado identificadas pelo navegador por mês.	EP e RC
7 - Intervenção†	Número de encaminhamentos/intervenções específicas oferecidas aos pacientes navegados por mês.	EP e RC
8 - Orientação sobre estudos clínicos	Número de pacientes que receberam orientação sobre estudos clínicos pelo navegador por mês.	EP e RC
9 - Encaminhamentos para estudos clínicos	Número de pacientes navegados por mês encaminhados ao departamento de estudos clínicos.	EP e RC
10 - Educação do paciente	Número de encontros realizados pelo navegador por mês para educação do paciente.	EP, RC e RDI
11 - Do diagnóstico ao tratamento inicial	Tempo transcorrido em dias entre o diagnóstico (data do resultado do anatomopatológico) e a modalidade de tratamento inicial (data do primeiro tratamento).	EP e RC
12 - Do diagnóstico à primeira consulta de oncologia	Tempo transcorrido em dias entre o diagnóstico (data do resultado do exame anatomopatológico) e a primeira consulta oncológica (data da primeira consulta).	EP e RC

*Definição de barreiras ao cuidado: Obstáculos que impedem um paciente com câncer de ter acesso a cuidados, serviços, recursos e/ou apoio. † Definição de intervenção: O ato de intervir, interferir ou interceder com a intenção de modificar o desfecho.

Domínio 3: Empoderamento/Defesa do Paciente		
Número e Métrica	Definição	Área de Impacto: Experiência do Paciente (EP) Resultados Clínicos (RC) Retorno do Investimento (RDI)
13 - Metas do paciente	Porcentagem de casos analisados por mês em que as metas do paciente foram identificadas e discutidas com o enfermeiro navegador.	EP,RC e RDI
14 - Apoio ao cuidador	Número de necessidades/preferências do cuidador discutidas com o navegador por mês.	RC
15 - Identificação do estilo de aprendizagem*	Número de pacientes navegados por mês cujo estilo de aprendizagem foi discutido durante o processo de acolhimento.	EP e RC

*Estilos de aprendizagem: • Visual (espacial): Prefere usar fotos, imagens e compreensão espacial • Auditivo (auditivo-musical): Prefere usar som e música • Verbal (linguístico): Prefere usar palavras faladas e escritas • Físico (cinestésico): Prefere usar seu corpo, mãos e tato • Lógico (matemático): Prefere usar lógica, raciocínio e sistemas • Social (interpessoal): Prefere aprender em grupos • Solitário (intrapessoal): Prefere trabalhar e estudar sozinho.

Domínio 4: Avaliação/Apoio Psicossocial		
Número e Métrica	Definição	Área de Impacto: Experiência do Paciente (EP) Resultados Clínicos (RC) Retorno do Investimento (RDI)
Métrica 16 - Rastreamento do sofrimento psicossocial	Número de pacientes navegados por mês que foram rastreados para o sofrimento psicossocial em uma consulta médica fundamental* com uma ferramenta validada.†	EP e RC
Métrica 17 - Encaminhamentos para apoio social	Número de pacientes navegados encaminhados para redes de apoio social por mês.	EP,RC e RDI

*De acordo com a National Comprehensive Cancer Network (NCCN) os pacientes devem ser rastreados para o sofrimento em todas as consultas médicas como uma marca registrada do cuidado centrado no paciente.Minimamente os pacientes devem ser rastreados para o sofrimento na primeira consulta oncológica e com intervalos regulares, especialmente em alterações do estado da doença, como remissão, recorrência, progressão e complicações relacionadas ao tratamento. † Termômetro da angústia da NCCN (versão validada em português).

Domínio 5: Sobrevivência		
Número e Métrica	Definição	Área de Impacto: Experiência do Paciente (EP) Resultados Clínicos (RC) Retorno do Investimento (RDI)
Métrica 18 - Plano de cuidados de sobrevivência	Número de pacientes navegados por mês que receberam um plano de cuidados de sobrevivência ao término do tratamento oncológico.	EP e RC
Métrica 19 - Transição* do Tratamento para a Sobrevivência	Porcentagem de casos analíticos navegados por mês que passaram do tratamento oncológico concluído para a sobrevivência.	EP e RC
Métrica 20 - Encaminhamentos para equipe multidisciplinar na consulta de acompanhamento pós-tratamento oncológico	Número de pacientes navegados por mês encaminhados para equipe multidisciplinar na consulta de acompanhamento pós tratamento oncológico.	EP,RC e RDI

*Definição de transições de cuidado: O movimento que os pacientes fazem entre profissionais de saúde e ambientes à medida que a sua condição e necessidades de cuidados mudam durante o curso de uma doença crônica ou aguda.

continue...

...Continuation

Domínio 6: Cuidados Paliativos/Fim de vida		
Número e Métrica	Definição	Área de Impacto:<br>Experiência do Paciente (EP)<br>Resultados Clínicos (RC)<br>Retorno do Investimento (RDI)
Métrica 21 - Encaminhamentos para equipe de cuidados paliativos	Número de pacientes navegados por mês encaminhados para a equipe de cuidados paliativos.*	EP,RC e RDI

* A ASCO define cuidados paliativos como uma abordagem especializada que visa aliviar sintomas e promover qualidade de vida independentemente da fase da doença, podendo estar presente também no diagnóstico ou tratamento ativo.

Domínio 7: Funções e Responsabilidades Profissionais		
Número e Métrica	Definição	Área de Impacto:<br>Experiência do Paciente (EP)<br>Resultados Clínicos (RC)<br>Retorno do Investimento (RDI)
Métrica 22 - Conhecimento da navegação no momento da orientação	Porcentagem de novos contratados que concluíram as competências básicas de navegador desenvolvidas institucionalmente. *	RC
Métrica 23 - Revisão anual das competências básicas do navegador em oncologia	Porcentagem de profissionais que concluem anualmente o treinamento institucional de competências essenciais do navegador oncológico.*	RC

* Para programas de navegação compostos por enfermeiros navegadores considerar as competências normatizadas pelo Conselho Federal de Enfermagem (COFEN) com a Resolução de número 735 de 2024.

Domínio 8: Gestão de Operações, Desenvolvimento Organizacional e Economia da Saúde		
Número e Métrica	Definição	Área de Impacto:<br>Experiência do Paciente (EP)<br>Resultados Clínicos (RC)<br>Retorno do Investimento (RDI)
Métrica 24 - Taxa de readmissão em 30, 60 e 90 dias	Número de pacientes navegados readmitidos no hospital em 30, 60 e 90 dias.	RDI
Métrica 25 - Orçamento Operacional de Navegação*	Despesas operacionais mensais por item de linha.#	RDI
Métrica 26 - Número de casos navegados	Número de novos casos†, casos abertos‡, e casos encerrados§ navegados por mês.	RDI
Métrica 27 - Encaminhamentos para serviços geradores de receita	Número de encaminhamentos mensais pelo programa de navegação para serviços geradores de receita.	RDI
Métrica 28 - Taxa de não comparecimento	Número de pacientes navegados que não compareceram a uma consulta e/ou agendamento de tratamento agendado por mês.	RDI
Métrica 29 - Retenção de pacientes por meio da navegação	Número de casos analíticos por mês ou trimestre que permaneceram na instituição devido à navegação.	RDI
Métrica 30 - Uso do serviço de emergência	Número de pacientes navegados por mês que utilizaram o serviço de emergência.	RDI
Métrica 31 - Admissões de emergência por número de pacientes com terapia antineoplásica	Número de admissões de pacientes navegados por 1.000 pacientes com terapia antineoplásica por mês que passaram pelo serviço de emergência.	RDI

*Definição de orçamento operacional: A combinação de despesas conhecidas, custos futuros esperados e receitas previstas ao longo de um ano. † Definição de novos casos: Novos pacientes encaminhados ao programa de navegação por mês. ‡ Definição de casos abertos: Casos que permanecem aberto por mês. §Definição de casos encerrados: Número de casos encerrados por mês. Fechamento formal do caso de um paciente no programa de navegação. # Realizar análise comparativa correlacionando despesas operacionais para sustentabilidade do programa com os resultados atingidos em Experiência do Paciente (EP), Resultados Clínicos (RC) e Retorno do Investimento (RDI).

Domínio 9: Pesquisa, Qualidade e Melhoria de Desempenho		
Número e Métrica	Definição	Área de Impacto:<br>Experiência do Paciente (EP)<br>Resultados Clínicos (RC)<br>Retorno do Investimento (RDI)
Métrica 32 - Experiência do paciente/Avaliação do atendimento	Experiência do paciente ou resultados da pesquisa de satisfação do paciente por mês (utilização de ferramenta de navegação específica para referência interna).	EP
Métrica 33 - Validação do programa de navegação com base na avaliação das necessidades da comunidade	Monitoramento anual de um objetivo principal do programa de navegação, conforme definido pelo comitê de câncer. Exemplo: População atendida.	EP,RC e RDI
Métrica 34 - Transição de pacientes* desde a entrada institucional	Porcentagem de casos analisados por mês que passaram da porta de entrada institucional para a modalidade de tratamento inicial.†	EP e RC
Métrica 35 - Investigação diagnóstica	Número de dias entre a data de realização do exame diagnóstico até a data do resultado do anatomopatológico para pacientes navegados.	RC

*Definição de transição do cuidado: O movimento que os pacientes fazem entre profissionais de saúde e ambientes à medida que a sua condição e necessidades de cuidados mudam durante o curso de uma doença crônica ou aguda. † Definição de modalidade: medicamentoso, radioterapia e cirurgia.
